# Convolutional neural network for cell classification using microscope images of intracellular actin networks

**DOI:** 10.1371/journal.pone.0213626

**Published:** 2019-03-13

**Authors:** Ronald Wihal Oei, Guanqun Hou, Fuhai Liu, Jin Zhong, Jiewen Zhang, Zhaoyi An, Luping Xu, Yujiu Yang

**Affiliations:** 1 Open FIESTA Center, Tsinghua University, Shenzhen, P.R. China; 2 Center for Nano and Micro Mechanics, School of Aerospace Engineering, Tsinghua University, Beijing, P.R. China; 3 Graduate School at Shenzhen, Tsinghua University, Shenzhen, P.R. China; Pavol Jozef Safarik University in Kosice, SLOVAKIA

## Abstract

Automated cell classification is an important yet a challenging computer vision task with significant benefits to biomedicine. In recent years, there have been several studies attempted to build an artificial intelligence-based cell classifier using label-free cellular images obtained from an optical microscope. Although these studies showed promising results, such classifiers were not able to reflect the biological diversity of different types of cell. While in terms of malignant cell, it is well-known that intracellular actin filaments are altered substantially. This is thought to be closely related to the abnormal growth features of tumor cells, their ability to invade surrounding tissues and also to metastasize. Therefore, being able to classify different types of cell based on their biological behaviors using automated technique is more advantageous. This article reveals the difference in the actin cytoskeleton structures between breast normal and cancer cells, which may provide new information regarding malignant changes and be used as additional diagnostic marker. Since the features cannot be well detected by human eyes, we proposed the application of convolutional neural network (CNN) in cell classification based on actin-labeled fluorescence microscopy images. The CNN was evaluated on a large number of actin-labeled fluorescence microscopy images of one human normal breast epithelial cell line and two types of human breast cancer cell line with different levels of aggressiveness. The study revealed that the CNN performed better in the cell classification task compared to a human expert.

## Introduction

Cell classification is of great importance to medical diagnosis, personalized treatment and disease prevention. Yet, classifying different types of cell with high precision has remained a challenging task, given difference in shape and size, and also some impacts caused by external environment. In addition, variations in illumination also cause the contrast between cell boundary and background to differ. All of these can have detrimental effects on the accuracy of cell classification.

Fortunately, machine learning has been developing rapidly as an important instrument for such difficult task recently, including in the field of biology and medicine [1]. It has been used for genomic data analysis [2], medical images analysis [3], analysis of tissue specimens [4] and even cell classification based on cell images [5, 6]. However, these cell classification tasks were only done based on bright-field microscopy images, which were not able to reflect the biological diversity of different cell types.

Cytoskeleton is a system of filaments and fibers that exists in the cytoplasm of eukaryotic cells. It provides cells the ability to maintain their shape and internal organization. Furthermore, it also plays an important role as a mechanical support that allows cells to perform essential functions, including cell signaling, division and movement. The cytoskeletal matrix is a dynamic structure made of microfilaments (actin filaments), intermediate filaments and microtubules. Among them, alterations in actin filaments are thought to be closely related to the development of cancer [7–11]. Actin filaments are composed of identical actin proteins arranged in long spiral chains. It has an important role in maintaining cell shape and movement. In malignant cells, actin filaments are known to be altered substantially [10]. Therefore, cancer cells have different mechanical properties from normal and benign cells. This phenomenon is believed to be responsible of the ability of the cells to migrate to nearby tissues through blood or lymph vessels and to form secondary tumors at distant sites [12]. Therefore, structure and organization of actin filaments may be a potential source of information about the biological behavior of cell.

However, despite can provide such valuable information, these features can hardly be detected by human eyes. Furthermore, cell classification based on some specific subcellular features, actin filaments organization in particular, are not yet widely covered by machine learning. This is partially due to the lack of large dataset to support its application. Yet, the application of machine learning in this field would be promising, given the ability to analyze actin filaments organization is potential for providing valuable information regarding the biological behavior of cell and the major difficulty for human to manually detect these features.

In this study, we collected a large dataset of more than 1500 cells with visualized actin filaments network. Furthermore, we demonstrated a successful application of convolutional neural network (CNN) in cell classification of one normal breast epithelial cell line MCF-10A (non-aggressive) and two breast cancer cell lines, MCF-7 (less aggressive) and MDA-MB-231 (more aggressive), based on these images.

## Materials and methods

### Cell culture

One non-cancerous human breast epithelial cell line (MCF-10A) and two cancerous human breast epithelial cell lines (MCF-7 and MDA-MB-231) obtained from American Type Culture Collection (ATCC, Rockville, MD, USA) were chosen for the experiments. It is well known that MCF-7 is a less aggressive cancer cell line, while MDA-MB-231 is a more aggressive one [13]. MCF-10A cell line was grown in mammary epithelial cell growth medium (MEBM/MEGM, Lonza, Walkersville, MD, USA) supplemented with 4 μL/mL bovine pituitary extract (Lonza), 1 μL/mL human epidermal growth factor (Lonza), 1 μL/mL insulin (Lonza), 1 μL/mL hydrocortisone (Lonza) and 100 ng/mL cholera toxin (Sigma-Aldrich, St. Louis, MO, USA). MCF-7 cell line was cultured in advanced minimum essential medium (Thermo Fisher Scientific, Waltham, MA, USA) supplemented with 10% fetal bovine serum (FBS, Thermo Fisher Scientific). MDA-MB-231 cell line was maintained in Leibovitz’s L-15 (Thermo Fisher Scientific) supplemented with 10% FBS (Thermo Fisher Scientific). Both MCF-10A and MCF-7 cell lines were grown at 37 °C in 5% CO_2_, while MDA-MB-231 was maintained at 37 °C without CO_2_ in a humidified incubator.

### Acquisition of confocal immunofluorescence microscopy images

All cell lines were cultured for 4–5 passages after being defrosted prior to immunostaining. Cells were seeded at a density of 1 x 10^5^ cells/mL onto 12-well cluster plate and incubated for 24 hours. The cell monolayer was washed twice with phosphate buffered saline (PBS), then fixed with 3% paraformaldehyde in PBS for 10 minutes and permeabilized with 0.1% Triton X-100 in PBS for 3–5 minutes. For immunostaining, the cells were first blocked with 3% bovine serum albumin (BSA) in PBS for 20–30 minutes and then the filamentous actin (F-actin) was stained with Alexa Fluor 488 Phalloidin (Thermo Fisher Scientific) diluted 1:40 in PBS containing 3% BSA for 20 minutes in room temperature. Following washing with PBS, cells were then examined using Olympus FLUOVIEW FV1000 confocal laser scanning microscope (Olympus, Shinjuku, Tokyo, Japan) with a 60x Plan-Apochromat oil-immersion objective (NA = 1.42). Lastly, ImageJ2 (National Institutes of Health, USA) was used to analyze the images.

### Data pre-processing

#### Image enhancement

**Fig 1:**
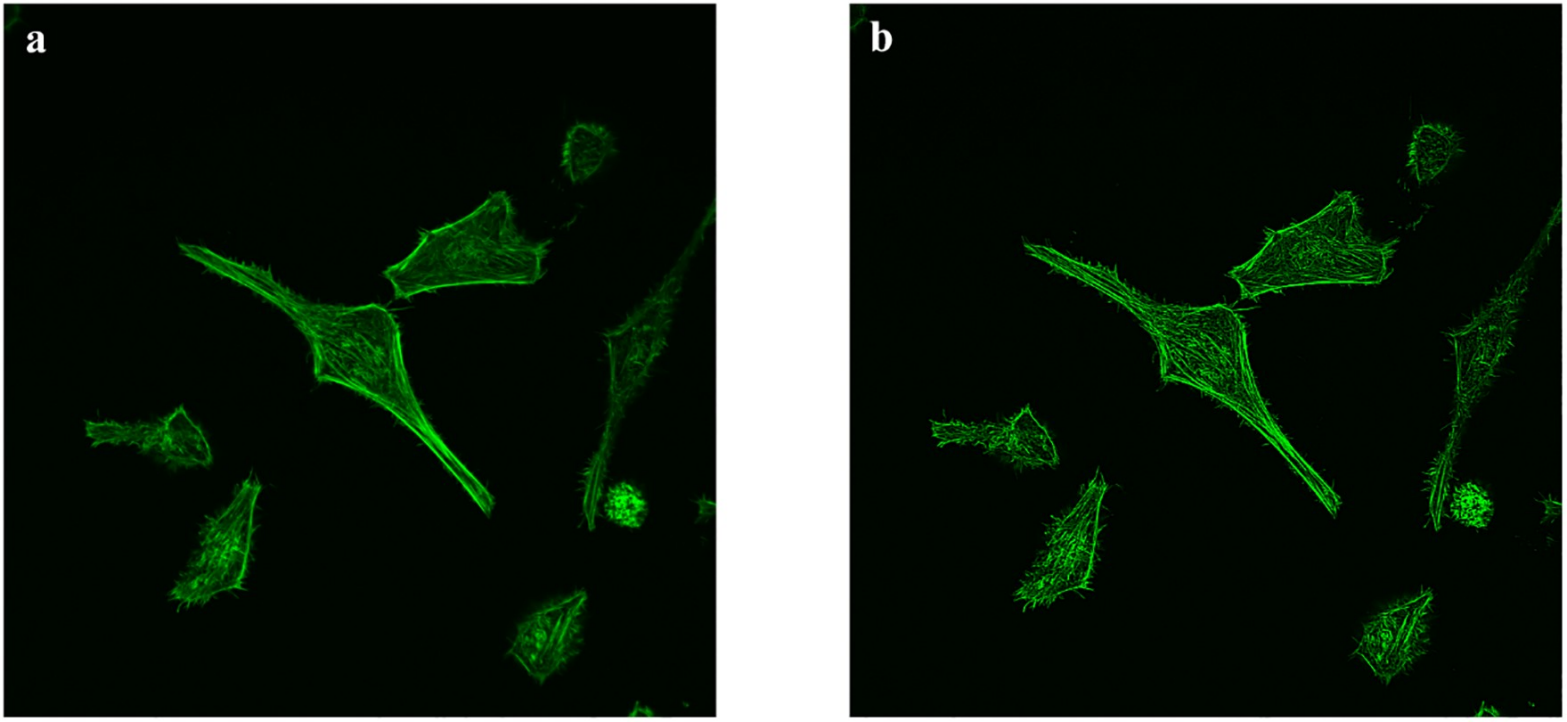
The comparison between image before (a) and after (b) image enhancement.

Current image enhancement methods, such as edge and contrast enhancement, are considered to be helpful for human eyes. However, its advantages in computer vision and machine learning have not been confirmed. Our study made comparison of two different models to reveal whether or not image enhancement techniques (using edge enhancement filters from Python Imaging Library/Pillow) would improve the performance of the network. Fig 1 shows the comparison of the image before and after image enhancement. The convolution matrix used by Pillow for the image filters is as follows:

$$\left[\begin{array}{ccc} -1 & -1 & -1\\ -1 & 10 & -1\\ -1 & -1 & -1 \end{array}\right]$$

#### Data augmentation

It is believed that data augmentation can improve accuracy and help to reduce overfitting [14]. In addition, data augmentation was crucial in the success of CNN [15]. Even more, the approach has also been proved to be advantageous for cell image classification task [16]. Thus, similar step was also implemented in our study by performing four different rotations, cropping and flipping on each image in the training set, resulting in a significant increase in the number of the images.

### Convolutional neural network

Firstly, images of three different types of cell were divided into training set and test set with a ratio of 10:1. After data augmentation was implemented, 3-fold cross-validation was then applied. The CNN was built according to the VGG-16 architecture [17]. The network consisted of 13 convolutional layers, 5 max-pooling layers and 2 fully connected layers. Lastly, a softmax layer containing 3 neurons was applied to classify the cells into three different classes: MCF-10A (non-aggressive), MCF-7 (less aggressive) and MDA-MB-231 (more aggressive).

**Fig 2:**
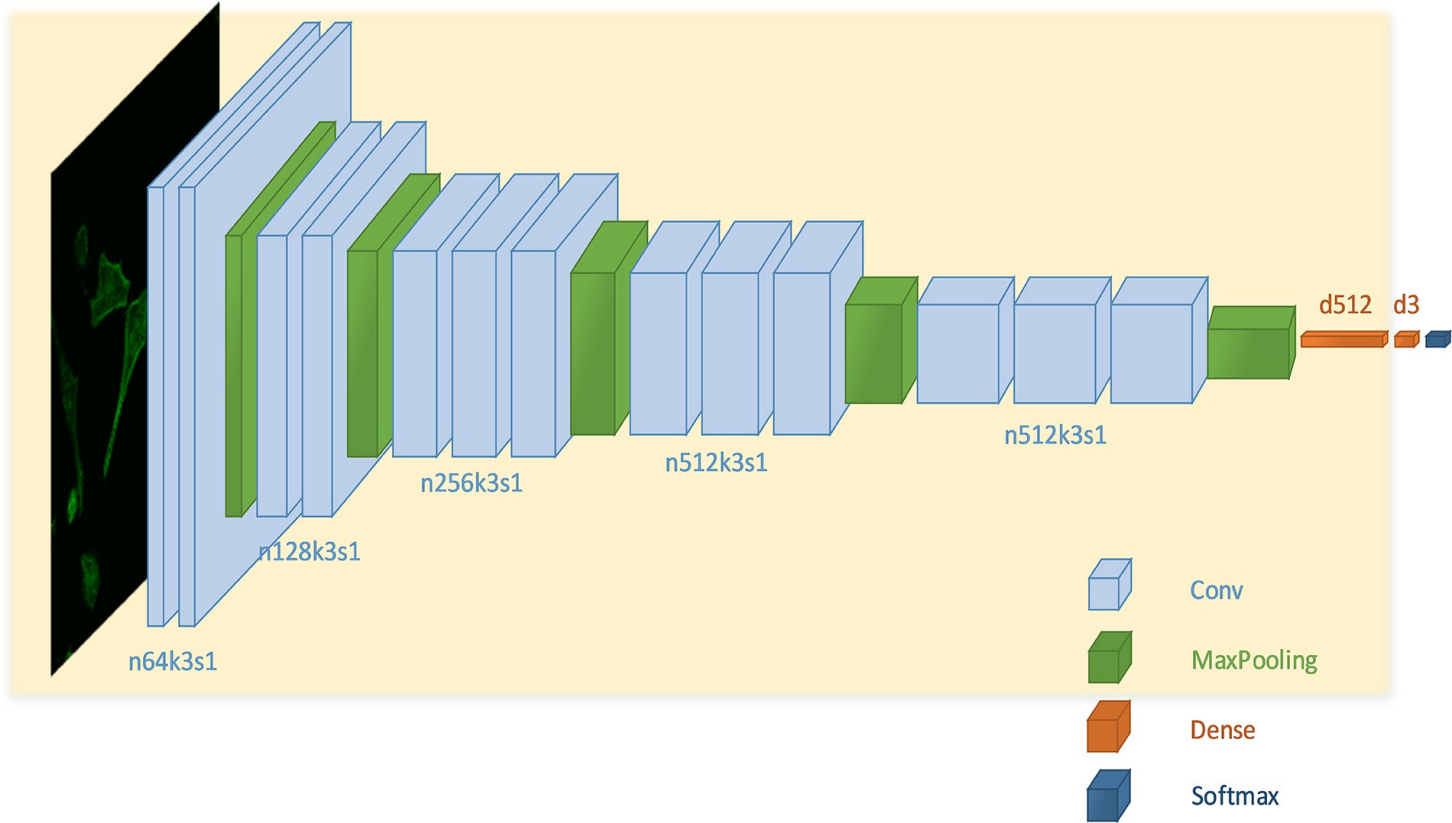
The architecture of the convolutional neural network with corresponding kernel size (k), number of feature map (n) and stride (s) indicated for each convolutional layer and number of unit for each dense layer.

The input image resolution was set to 64x64 pixels and the activation function used was ReLU. Following each activation function, BatchNormalization was added for regularization. Furthermore, dropout was performed in order to constrain the fully connected layers and further reduce overfitting. The topology of the network is shown in Fig 2.

The network parameters used were as follows:

1. Optimizer: “Adam”;
2. Learning rate: 0.001;
3. L2 regularization term: 0.0005;
4. Dropout rate: 0.4/0.5;
5. Batch size: 4.

The loss function was set to the cross-entropy cost function:

$$L=-\frac{1}{n}\sum_{x}\left[y\;\text{ln}\;a+(1-y)\text{ln}\left(1-a\right)\right]$$

Where y was the desired output and a was the actual output of the neurons. Above loss function can overcome the problem of slow weight update without harming the classification performance.

### Transfer learning

As stated before, transfer learning is the fine-tuning process of neural network model which is pre-trained on other huge amount of data. The first 14 layers of our CNN was pre-trained on the ImageNet classification dataset (ILSVRC2012) (shaded in light yellow in Fig 2). The images were first cropped from the original size of 224x224 pixels down to 64x64 pixels before the pre-training was begin. On top of it, one task-specific fully connected layer with random initialized weights was attached. Finally, three different cell types were classified using softmax function. All layers were thoroughly fine-tuned on our dataset.

## Results

### Cytoskeleton features of the cell lines

**Fig 3:**
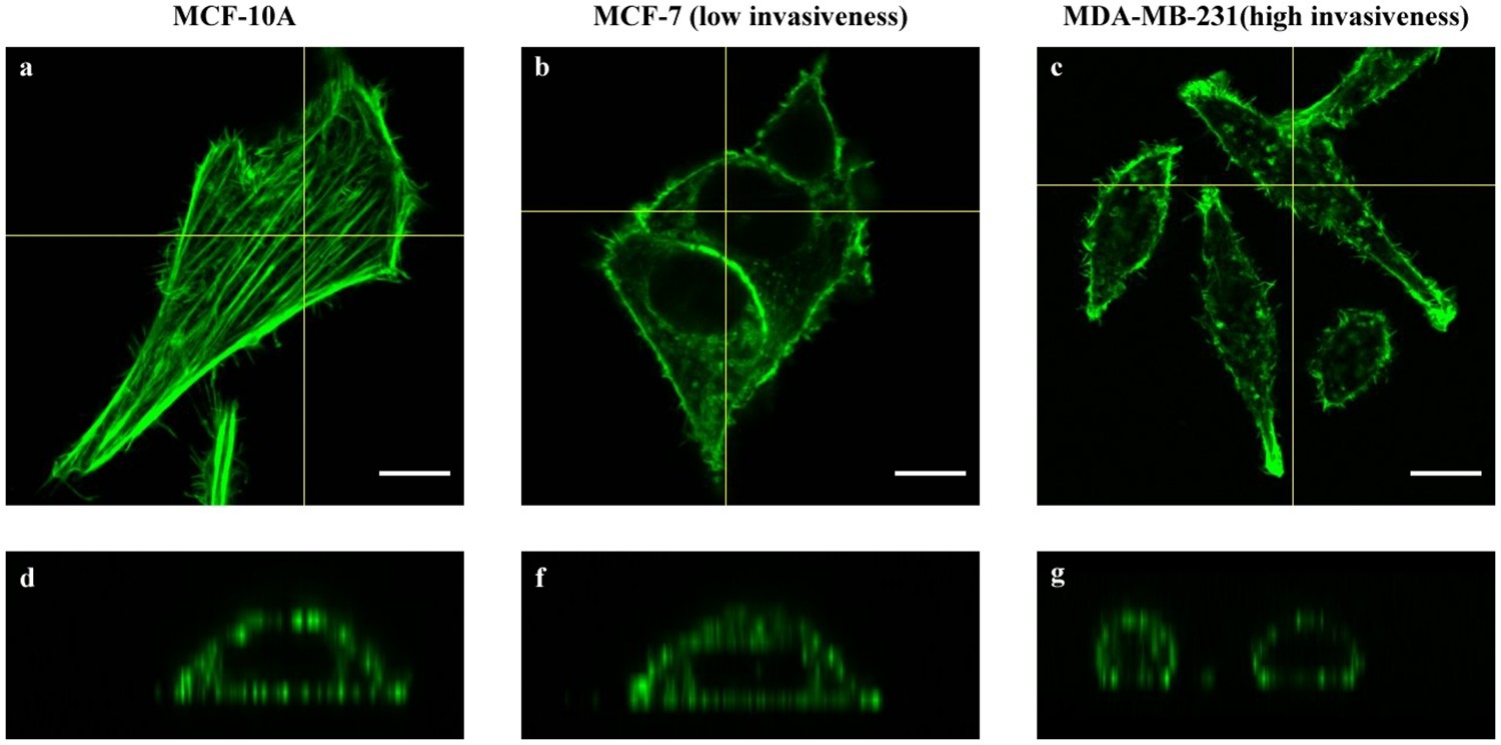
Z-stack actin-labeled fluorescence microscopy images and the orthogonal projections of MCF-10A (a,d), MCF-7 (b,e) and MDA-MB-231 (c,f).

**Fig 4:**
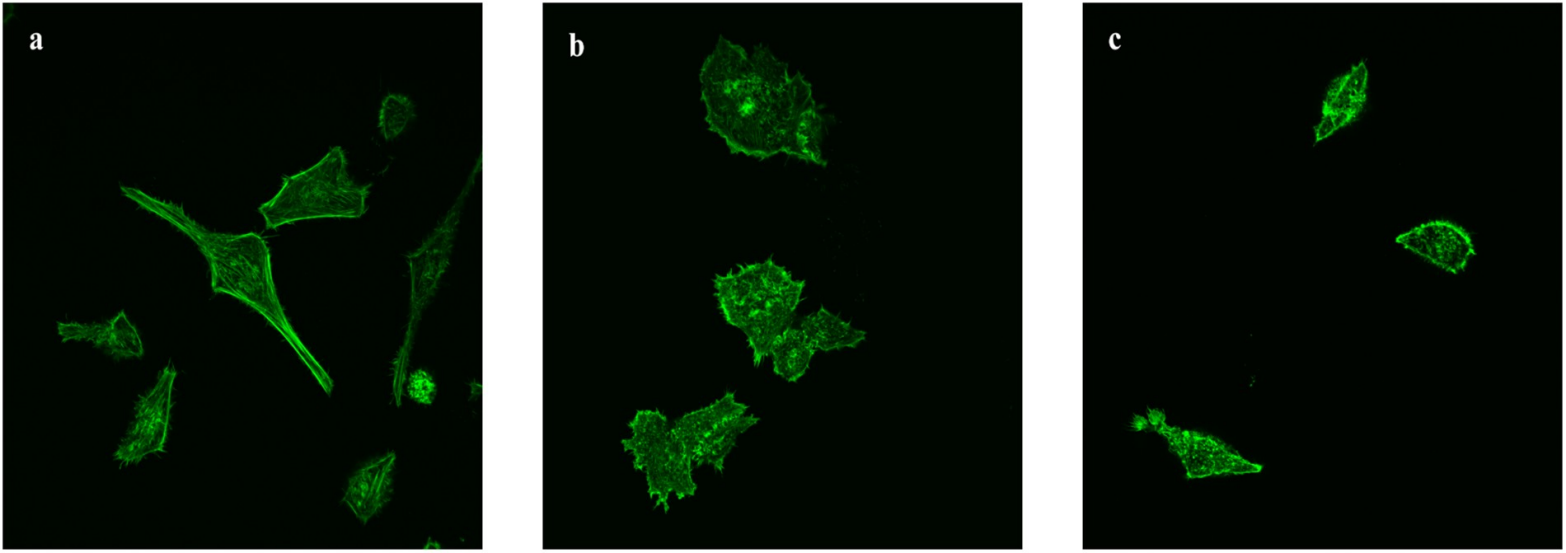
Actin-labeled fluorescence microscopy images of MCF-10A (a), MCF-7 (b) and MDA-MB-231 (c).

The difference in cytoskeleton structures between three cell lines were observed during the acquisition of the images (Figs 3 and 4). In term of MCF-10A, actin were either arranged in short and widespread stress fibers or long and well-aligned bundles. Notably, actin filaments were distributed evenly throughout the cell body. In contrast, the two cancer cell lines exhibited less prominent actin filaments structures. Diffuse distribution of actin filaments was observed in the cytoplasm which might be the reason of weaker localization in the cortical cytoskeleton. Furthermore, the scarce amount of long actin stress fibers detected in the two cancer cell lines were mainly located in the cell borders or ventral regions, while no long actin bundles were found in the cortical regions. The thickness of actin filament was also measured from ten images of each cell line, which were 0.5±0.1 μm for MCF-10A and 0.4±0.1 μm for the two cancer cell lines. However, this was only a qualitative evaluation considering these figures are very close to the resolution limit of the confocal microscope. Moreover, even though the difference in thickness may be just the inherent characteristics of the cell lines, notable reduction in the thickness of actin stress fibers was observed in different types of cancer cell [18–21].

Orthogonal projections were constructed in order to provide three-dimensional understanding of the distribution of actin filaments within the three cell lines (Fig 3d, 3e and 3f). As can be seen, the surface of MCF-10A cell line contained more actin filaments than the two cancer cell lines. It was mainly found in the basal regions adjacent to the substrate and in the apical regions close to the higher perinuclear areas. In contrast, only scarce amount of actin filaments were found in the perinuclear apical areas of both cancer cell lines, especially for MDA-MB-231 cell line (highly invasive). Rather, unorganized actin networks dominated the cytoskeleton organization in these regions and actin bundles almost exclusively existed in the lower basal areas.

In summary, a total of 552 images were captured, in which each image contained at least three individual cells, resulting in more than 1500 individual cell images (approximately 500–600 individual cell images for each type of cell). To be specific, MCF-10A, MCF-7 and MDA-MB-231 classes each contained 182, 186 and 184 images, respectively.

### Evaluation of human expert performance

In order to make a comparison between the performance of the chosen algorithm and that of human expert, one researcher in our institution who was experienced in actin filaments-related studies was asked to carry out the classification task on our test set containing of 52–100 images from each class.

The confusion matrix is provided in Table 1. To summarize, the accuracy of the classification task done by the human expert was 78.6%. Further analysis of the results revealed that misclassification often arose from fragmented and blurred images which cannot be avoidable in current imaging technology.

**Table 1:** Confusion matrix of cell classification task performed by a human expert.

Human guess	True class		
	MCF-10A	MCF-7	MDA-MB-231
MCF-10A	73.0%	3.9%	23.1%
MCF-7	2.9%	91.2%	5.9%
MDA-MB-231	24.0%	3.0%	73.0%

### Convolutional neural network performance

Before applying data augmentation, the dataset were divided into training set and test set with a ratio of 10:1. The test set contained 12 images of MCF-10A cell line, 16 images of MCF-7 cell line and 24 images of MDA-MB-231 cell line. After data augmentation was performed, the number of the images in the training set became eight times bigger than before, containing 1360 images of each MCF-10A and MCF-7 cell lines and 1280 images of MDA-MB-231 cell line. Three-fold cross-validation was applied for better assessment of the methods. The network was trained using Keras framework [5] on Nvidia GeForce GTX TITAN Z GPU with 12 GB of GPU memory. Four models were performed, which were:

1. Model without image enhancement and transfer learning;
2. Model without image enhancement, but with transfer learning;
3. Model with image enhancement (edge enhancement), but without transfer learning;
4. Model with image enhancement (edge enhancement) and transfer learning.

A thousand epochs of training were carried out for each model. The training time is listed in Table 2. The accuracy of model b and d reached an accuracy of more than 95% within just ten epochs, compared to model a and c that reached an accuracy of 90% after around 200 epochs.

**Table 2:** The training time of four models.

Model	a	b	c	d
Time (hours)	6.3	3.7	6.4	4.4

**Fig 5:**
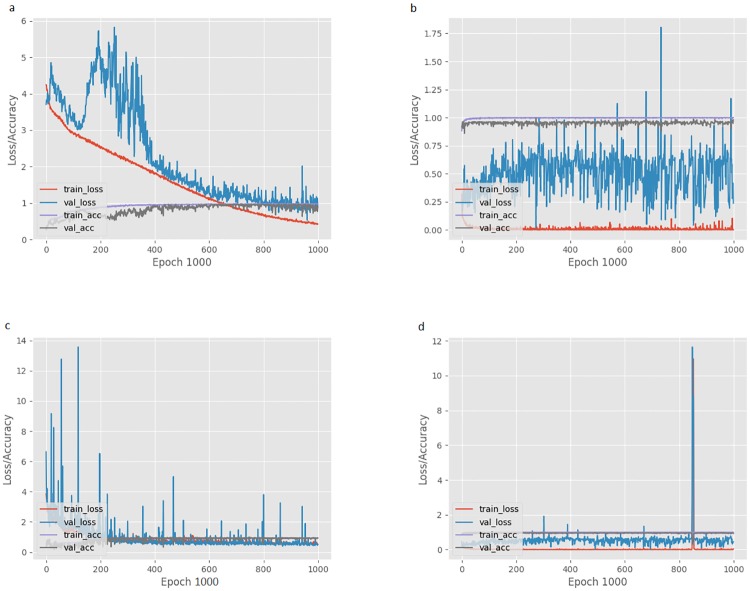
The training and validation loss of the classifier after 1000 epochs of training using four different methods. (a) Model without image enhancement and transfer learning; (b) Model without image enhancement, but with transfer learning; (c) Model with image enhancement (edge enhancement), but without transfer learning; (d) Model with image enhancement (edge enhancement) and transfer learning. *Abbreviations*: train_loss, training loss; val_loss, validation loss; train_acc, training accuracy; val_acc, validation accuracy.

**Fig 6:**
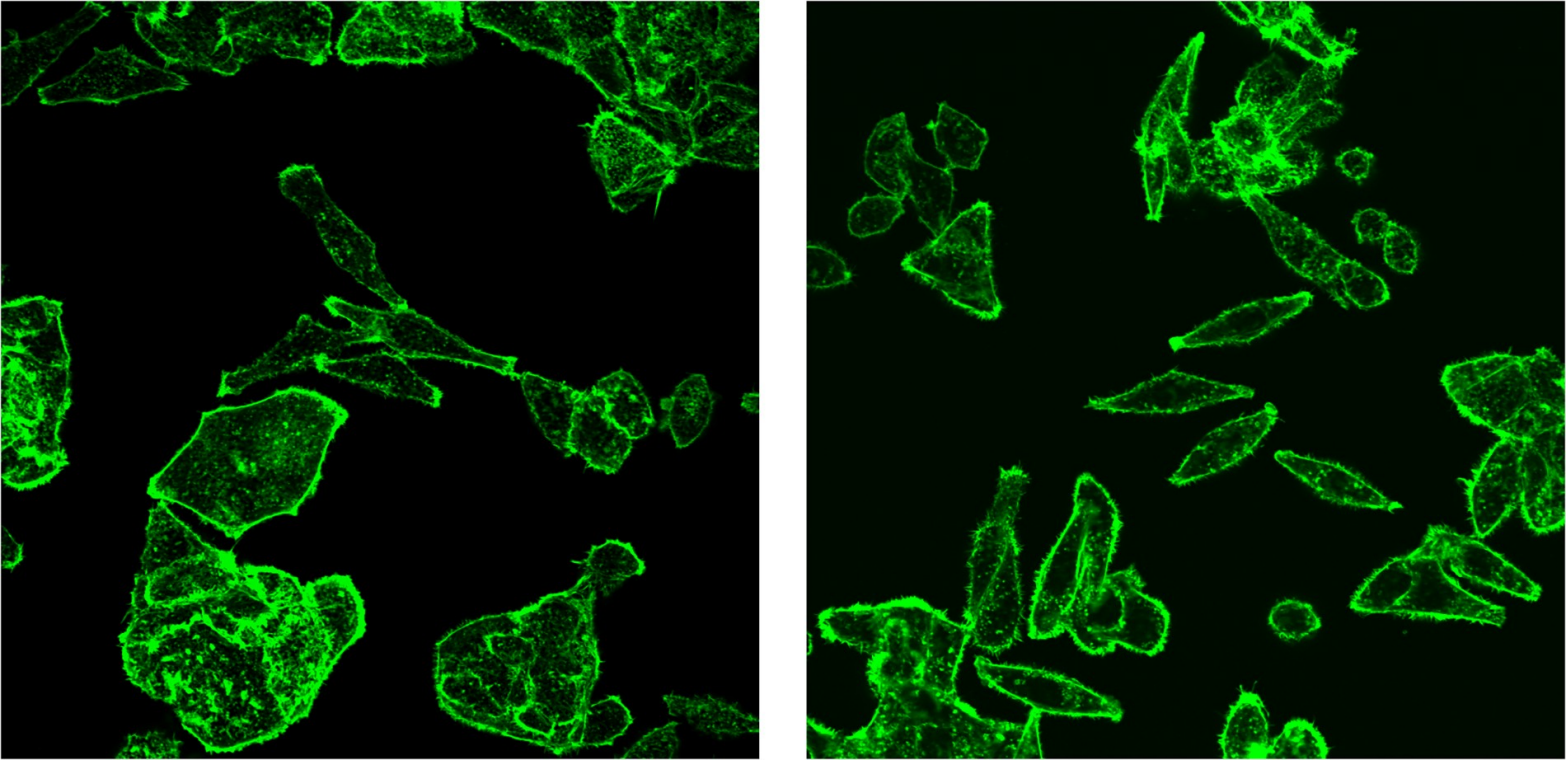
The two immunofluorescence images of MDA-MB-231 cell line in the test set which were misclassified as MCF-10A cell line by the network.

The accuracy of four models after 1000 epoch are shown in Table 3. The best classification result was obtained using model b, which is the pre-trained model without image enhancement. The confusion matrix of the network performance using model b after 1000 epochs of training is provided in Table 4. The training and validation loss of the classifier after 100 and 1000 epochs of training are shown in Fig 5. Furthermore, the only two images in the test set which were misclassified by the network are shown in Fig 6.

**Table 3:** The accuracy of four models on train and test sets.

Model	a	b	c	d
Train accuracy	98.4%	100.0%	93.8%	100.0%
Test accuracy	96.0%	97.2%	93.3%	94.1%

**Table 4:** Confusion matrix of cell classification task performed by the convolutional neural network using model b.

CNN guess	True class		
	MCF-10A	MCF-7	MDA-MB-231
MCF-10A	100.0%	0%	8.4%
MCF-7	0%	100.0%	0%
MDA-MB-231	0%	0%	91.6%

## Discussion

In our study, we analyzed the actin cytoskeleton structures of normal and cancer cells. We compared human-derived breast epithelial cell line MCF-10A with two human-derived breast cancer cell lines, MCF-7 and MDA-MB-231 and found substantial difference in the actin cytoskeleton structures between them. The latter two cancer cell lines are different in term of the status of three key receptors conventionally used for breast cancer subtyping, which are estrogen receptor (ER), progesterone receptor (PR) and human epithelial receptor 2 (HER2) [13]. MCF-7 is a breast cancer cell line with ER, PR positive and HER2 negative and therefore is categorized as less invasive, while MDA-MB-231 is a triple negative (ER, PR and HER2 negative) and is considered to be more invasive [13]. Though the genetic and molecular origins of malignancy are various, difference in the mentioned figures above during the development of the diseases are common in many types of cancer [18–20, 22, 23].

Mechanical features of cancer cells are thought to have an important role during metastasis, which is a process of active migration of the cells to the nearby or distant tissues. This action involves a vital change in the formation of the cytoskeleton [24]. Even though the mechanism behind the reorganization of actin filaments in cancer cells has not been well understood, the difference in actin filaments structures between normal and cancer cells may reflect the biological behavior of the cells and serve as an extra diagnostic marker. Unfortunately, these features cannot be precisely detected by human eyes as proved in our study.

Therefore, we proposed a CNN-based analysis and classification method which then provides further confirmation of the superiority of CNN upon human in performing the task (97.6% vs 78.6%), especially on the images which quality were not good enough due to the limitation of current imaging technology. Through the comparison made between the four different models applied, we also found that transfer learning is a powerful method to improve the performance of CNN, especially on a small dataset. Our results showed that the training accuracy reached 100% when transfer learning was applied. Moreover, we proved that existing image enhancement methods are designed only to improve the perceptual quality of an image for human observers, but not for CNN, resulting in no improvement seen on the performance of the CNN after applying the methods. Lastly, according to the confusion matrices in both human expert- and CNN-based classification, we can observe that misclassification between MCF-10A cell line and MDA-MB-231 cell line (more aggressive) were more frequently seen, compared to that of MCF-10A cell line and MCF-7 cell line (less aggressive). We assumed that it was mainly because there was similarity in the morphology of both MCF-10A and MDA-MB-231 cell lines. Therefore, both human expert and CNN might to some extent take into account the morphology of the cells in the classification process, which were inevitable. However, based on the confusion matrices too, we believed that the difference in F-actin structures was still the main consideration in both human expert and CNN.

CNN is currently considered as the basis of many state-of-the-art image analysis tasks [14]. It was inspired by the structure of animal visual cortex [25]. It consists of various consecutive steps, including convolutional, non-linearity and pooling layers. These are then followed by other convolutional and fully connected layers. Raw pixel intensity image acts as the input of the network, whereas the output layer comprises a number of neurons in which each neuron corresponds to one class. Weights in CNN are optimized by reducing the classification error on training set through backpropagation algorithm [26].

In the field of biomedical image analysis, CNN has shown a success in breast cancer histology image analysis [27]. Rakhlin et al [27] utilized various deep convolutional neural networks for feature extraction, including pre-trained ResNet-50, InceptionV3 and VGG-16 networks, and a fast, distributed and high-performance gradient boosted tree classifier, Light GBM. The dataset contained a total of 400 hematoxylin and eosin stained breast histology microscopy images of four balanced classes: normal, benign, in situ carcinoma and invasive carcinoma [27]. The approach reached an average accuracy of 87.2 ± 2.6% in 4-class classification and 93.8 ± 2.3% in 2-class non-cancer (normal and benign) vs. cancer (in situ and invasive) classification, which outperformed other similar solutions [27].

Apart from that, CNN has also been successfully implemented in the field of bioinformatics. Zhang et al [28] proposed a novel deep learning architecture to enhance the robustness of protein secondary structure prediction by integrating CNN, bidirectional recurrent neural network and residual network. The study showed a superior performance compared to previous state-of-the-art methods [28].

The application of CNN in biomedical image analysis is currently hindered due to insufficient data. However, excellent performance can also be obtained even when only small dataset is available by using a pre-trained model on a large and diverse image dataset, which is called “transfer learning” [29]. The method is powerful for training a network on fewer data or when key resources are limited. Since the features of early CNN layers are generic, it can be adapted flexibly to perform various tasks [29], such as CNN pre-trained on ImageNet [30] and later was used to analyze various medical images, such as CT, ultrasound and X-ray datasets. Also, transfer learning can subsequently reduce overfitting on small datasets while boosting performance through fine-tuning.

Our study accelerates the implementation of CNN in biomedical areas by collecting a large dataset of actin-labeled fluorescence microscopy images, which is to the best of our knowledge is the largest dataset in the field. Furthermore, we used human-derived cells in order to strengthen the clinical value of our study. The images were then used to train a CNN which performed cell classification task. We believe that this is the first work of using intracellular features which can reflect the biological behavior of cell in order to classify different types of cell. Future improvements can be made to enhance the current methods, including but not limited to: 1) to collect more images of the three cell lines used and if possible, images of other breast cancer cell lines; 2) to apply image segmentation so the network can segment every single cell in the images before learning its features during the training process. In the future, we will also try to build similar network in order to recognize different levels of drug exposure targeting intracellular features of cells.

Still, this study also had several limitations. First, only three cell lines are included in the study due to the huge amount of time spent on the image acquisition. Next, since the cancer tissue derived from patients usually contains different types of cell, our study results cannot be directly implemented into clinic. However, recent advances in imaging approach are currently increasing the speed of image acquisition and significantly improving the image quality at the same time. This certainly will create more opportunities of implementing CNN even further in the field of biomedicine. Therefore, our study rather gives the feasibility of performing classification task based on subcellular features, which perhaps can provide more information regarding the biological diversity of cells. Furthermore, we have proved the advantage of transfer learning in improving network performance, especially when only limited data is available, and confirmed that current image enhancement techniques do not help, even harm network performance.

## Conclusion

In our study, we found that the actin stress fibers were scarce in the cancer cell lines compared to the normal one, which may promote invasion and the occurrence of metastasis. Thus, actin organization may reflect the biological behavior of cells. Unfortunately, human eyes are not well enough in analyzing the features and therefore, we proposed a CNN-based method to classify human breast-derived cell lines based on their actin filaments organization. A large dataset of actin-labeled fluorescence microscopy images was captured in order to train the network. Our results showed that the CNN applied outperformed the human performance in term of accuracy and also revealed that the image enhancement technique applied did not improve, even lower the performance of the network. Lastly, we also confirmed the advantage of transfer learning in improving network performance when only limited data are available.
